# Second-trimester serum high mobility group box-1 and uterine artery Doppler to predict preeclampsia

**DOI:** 10.1038/s41598-022-10861-1

**Published:** 2022-04-27

**Authors:** Varangkana Wairachpanich, Vorapong Phupong

**Affiliations:** Placental Related Diseases Research Unit, Department of Obstetrics and Gynecology, Faculty of Medicine, Chulalongkorn University, Rama IV Road, Pathumwan, Bangkok, 10330 Thailand

**Keywords:** Biomarkers, Diseases

## Abstract

The objective of this study was to identify the predictive value for preeclampsia of second-trimester serum high mobility group box-1 (HMGB1) and uterine artery Doppler in singleton pregnancies. Between April 2020 and April 2021, a prospective study was conducted on singleton pregnancies with a gestational age of 16–20^+6^ weeks at King Chulalongkorn Memorial Hospital, Department of Obstetrics and Gynecology, Faculty of Medicine, Chulalongkorn University, Bangkok, Thailand. Maternal characteristics, uterine artery Doppler, and serum HMGB1 were collected. Serum HMGB1 levels and mean uterine artery pulsatility index (UAPI) were combined to calculate the predictive value for preeclampsia. A total of 393 pregnant women were analyzed, with 25 cases (6.4%) developing preeclampsia and 5 cases (1.3%) developing early-onset preeclampsia. Baseline characteristics of preeclampsia and normal pregnant women were comparable. Preeclamptic pregnant women had significantly higher mean serum HMGB1 levels than normal pregnant women (1112.8 ± 363.1 ng/mL vs 910.8 ± 486.1 ng/mL, p = 0.013). There was no difference in the mean UAPI. Any early-diastolic notching was found more frequently in the preeclampsia group (32.0% vs 12.5%, p = 0.013). The cut-off value for serum HMGB1 levels above 1.04 MoM as abnormal value to predict preeclampsia had sensitivity, specificity, positive predictive value (PPV) and negative predictive value (NPV) of 88.0%, 53.5%, 11.4% and 98.5%, respectively. When using abnormal serum HMGB1 levels combined with mean UAPI above 95th percentile, the sensitivity, specificity, PPV and NPV to predict preeclampsia were 88.0%, 50.8%, 10.8% and 98.4%, respectively. This study showed that serum HMGB1 at 16–20^+6^ weeks of gestation were effective in predicting preeclampsia. The addition of UAPI did not improve the prediction performance.

## Introduction

Preeclampsia is a hypertensive pregnancy condition characterized by a new-onset of hypertension after 20 weeks of gestation, proteinuria, and, in some cases, significant end-organ injury^1^. Preeclampsia is still the second most frequent obstetrical complication, resulting in high maternal and neonatal morbidity and mortality^2,3^. According to King Chulalongkorn Memorial Hospital's obstetrical data records, the annual incidence of preeclampsia was 4.99% from 2015 to 2019, which was comparable to the global incidence^4^.

Preeclampsia is thought to be caused by various mechanisms, including poor placental implantation. The lack of trophoblastic invasion into the maternal deeper myometrial arterioles resulted in abnormal spiral artery remodeling^5,6^. Defective spiral arteries had higher resistance and lower oxygenation than normal spiral arteries, leading to placental ischemia, hypoxia, and necrosis. Anti-angiogenic factors and pro-inflammatory cytokines were induced by the necrosis of trophoblasts, which entered the maternal circulation and triggered systemic endothelial cells, contributing in clinical manifestations of preeclampsia several weeks later^7,8^.

A number of studies have found that abnormal spiral artery remodeling is linked to high resistance in the uterine artery^9^. Preeclampsia and poor pregnancy outcomes have been associated with an increase uterine artery Doppler impedance flow or early-diastolic notching waveforms^10,11^. However, uterine artery Doppler as a single marker has limited sensitivity and accuracy in predicting preeclampsia, thus, various maternal biochemical markers were combined with uterine artery Doppler to improve diagnostic yields^12–15^.

High-mobility group box 1 (HMGB1) is a DNA-binding intracellular protein. The primary functions of HMGB1 are DNA replication, transcription, repair, and nucleosome stabilization^16^. When tissue hypoxia or necrosis occurred, HMGB1 was released into the extracellular space through active and passive pathways, triggering the inflammatory response via intracellular signaling^17^. The interaction of HMGB1 with transmembrane receptors such as Toll-like receptors (TLR)-2, TLR-4, TLR-9, and the receptor for advanced glycation end-products (RAGE) activated pro-inflammatory cytokines including tumor necrosis factor (TNF), interleukin (IL)-1, IL-6, and IL-8. As a result, a sterile inflammatory response to tissue damage occurs^18,19^.

HMGB1 and its receptors were found in high concentrations in the serum and trophoblastic cytoplasm of pregnant women with preeclampsia, particularly in those with severe symptoms and preeclampsia that began before 34 weeks of gestation^20–22^. HMGB1 was found to be released by hypoxic trophoblasts and to increase endothelial cell permeability in previous studies^23,24^. As a result, HMGB1 has been considered as a crucial role in the pathogenesis of preeclampsia.

HMGB1 levels were shown to be increasing between 16 and 22 weeks of gestation^25^, prior to the onset of preeclampsia, at the same time as an anatomical scan and uterine artery Doppler evaluation. HMGB1 has never been used to predict preeclampsia. Thus, the primary objective of this study was to determine the predictive value of HMGB1 and uterine artery Doppler in the second-trimester for preeclampsia in singleton pregnancy. The secondary objective of the study was to find the correlation between HMBG1 levels and uterine artery Doppler and adverse pregnancy outcomes.

## Materials and methods

This is a prospective observational study of singleton pregnancies who received antenatal care at King Chulalongkorn Memorial Hospital, Department of Obstetrics and Gynecology, Faculty of Medicine, Chulalongkorn University, Bangkok, Thailand, between April 2020 and April 2021. The inclusion criteria were singleton pregnant women aged 18–45 years old with a gestational age of 16–20^+6^ weeks. Women with active medical conditions (such as chronic hypertension, diabetes), autoimmune diseases, current aspirin using, on anti-coagulant or immunosuppressive drug therapy, and pregnant women with fetal structural or chromosomal defects were excluded. The study was approved by the institutional ethics review board, Faculty of Medicine, Chulalongkorn University. This study has been performed in accordance with the Declaration of Helsinki. Before being enrolled in the study, all participants gave written informed consent. All pregnant women who received antenatal care in that period were invited and included.

Data were collected from participants after they enrolled into the study, including maternal characteristics, uterine artery Doppler, and serum HMGB1 levels. Participants' characteristics, medical background, and obstetrics history were obtained through interviews and electronic medical records.

Transabdominal ultrasound was used for anatomical scanning, and the uterine artery Doppler was collected by the principal investigator using ultrasonographic machines (GE Voluson E10, GE Medical Systems, Zipf, Austria) with a 2.0–7.0 MHz convex probe. Both sides of the uterine artery were marked as crossing the external iliac arteries in the parauterine area of the lower uterine segment region using the color Doppler mode. Pulsed-wave Doppler was used to achieve uterine artery waveforms with an insonation angle < 30° and a peak systolic velocity greater than 60 cm/s. On each side, three identical consecutive waveforms were recorded. The mean uterine artery pulsatility index (UAPI), as well as, the presence or absence of notching were recorded. The mean UAPI more than 95th percentile of each gestation was defined as abnormal uterine artery Doppler^10^.

For the serum HMGB1 assay, 10 mL of venous blood was drawn from the patients, then the serum was obtained by centrifugation at 2500 rpm for 10 min and preserved at − 80 °C until the assay was performed. The serum HMGB1 levels were quantitated by using commercial sandwich enzyme-linked immunosorbent assay (ELISA) kits according to the manufacturer's instructions (E1635Hu; Bioassay Technology Laboratory, Shanghai, China). The minimum detectable dose of HMGB1 is 0.24 ng/mL. The ranges of intra- and inter-assay variation are less than 8 and 10%, respectively.

Data on maternal and neonatal outcomes were collected from hospital electronic medical records. Pregnancy outcomes included a presence or absence of preeclampsia, gestational age at delivery and pregnancy complications. The neonatal outcomes included birth weight, Apgar scores, and neonatal complications. Preeclampsia was defined as a blood pressure of 140/90 mmHg or higher on two occasions at least 4 h apart after 20 weeks of gestation, with proteinuria (at least 2+ on urine dipstick test, urine protein-creatinine ratio ≥ 0.3, or urine protein ≥ 300 mg/day) or in the absence of proteinuria, new-onset hypertension with the new onset of any of the following: thrombocytopenia, renal insufficiency, impaired liver function, pulmonary edema or new-onset headache unresponsive to medication and not accounted for by alternative diagnoses or visual symptoms^1^. Early-onset preeclampsia was defined as the onset of preeclampsia before 34 weeks of gestation^1,26^. Fetal growth restriction was defined as fetuses with an estimated fetal weight less than the 10th percentile for gestational age^27^.

The sample size was calculated using the predicted sensitivity of the HMGB1 and uterine artery Doppler prediction test of 80% and the incidence of preeclampsia at KCMH in the previous 5 years of 4.99%. For this study, 386 pregnant women were needed, with adjustments for a 20% loss rate.

### Statistical analysis

IBM SPSS Statistics for Windows, version 22 (IBM, Armonk, NY, USA) was used for statistical analysis. Data were reported as n (%) and mean ± standard deviation (SD). Chi-square test was used for comparing categorical data. Unpaired t-test and Mann–Whitney U test were used for comparing continuous data. The 95th percentile UAPI was calculated according to gestational age. Median of serum HMGB1 was calculated according to gestational age. Multiple of median of serum HMGB1 was calculated by serum HMGB1 of pregnant woman divided by median of serum HMGB1 at the same gestational age. A cut-off value of serum HMGB1 level was calculated using the receiver operating characteristic (ROC) curve. Statistical significance was considered when p value was less than 0.05.

## Results

**Figure 1:**
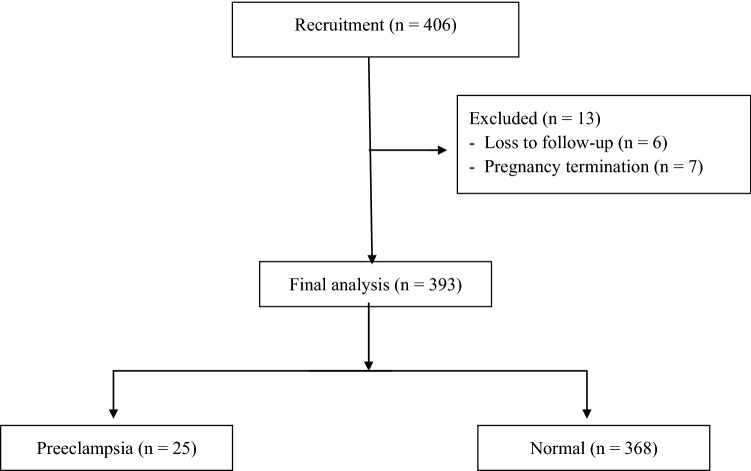
Recruitment flowchart for study participants.

A total of 406 pregnant women were recruited for the study, with 13 women were excluded due to loss to follow-up (n = 6), pregnancy termination (n = 7; 1 case with abortion; 4 cases of chromosomal abnormalities and 2 cases of major thalassemia disease) (Fig. 1). Data from 393 pregnant women were analyzed. Preeclampsia occurred in 25 of the patients (6.4%), with 5 cases (1.3%) had early-onset preeclampsia and 13 cases (3.3%) had preeclampsia with severe features.

Preeclamptic women and normal pregnant women had similar maternal age, gravida, parity, pre-pregnancy BMI, total weight gain, and gestational age at measurement. However, preeclamptic women had significantly higher mean arterial pressure than non-preeclamptic women (Table 1). Furthermore, preeclamptic women were more likely to have gestational diabetes, preterm delivery before 37 weeks of gestation, preterm delivery before 34 weeks of gestation, and fetal growth restriction. There were no differences in terms of Apgar score, length of stay, perinatal morbidity and mortality. As compared with normal pregnant women, newborns born to mothers with preeclampsia have a significantly lower birth weight and higher rate of low birth weight less than 2500*g* (Table 2).

**Table 1:** Baseline characteristics of women with and without preeclampsia.

	Without preeclampsia (n = 368)	With preeclampsia (n = 25)	p value
Maternal age (years)	34.0 ± 5.3	34.7 ± 5.4	0.560
Advanced maternal age (≥ 35 years old)	223 (60.6)	19 (76)	0.126
Primigravida	157 (42.7)	11 (44)	0.436
**Parity**			0.580
0	197 (53.5)	15 (60.0)	
≥ 1	171 (46.5)	10 (40.0)	
Pre-pregnancy BMI (kg/m^2^)	22.8 ± 3.9	24.4 ± 5.6	0.063
Obesity (BMI ≥ 30 kg/m^2^)	24 (6.5)	3 (12.0)	0.400
Total weight gain (kg)	13.2 ± 5.0	11.8 ± 6.6	0.182
Mean arterial pressure (mmHg)	80.2 ± 9.0	87.2 ± 11.3	< 0.001
GA at measurement (weeks)	18.3 ± 1.1	18.1 ± 1.2	0.444

Data are presented as the mean ± SD or as N (%).

*GA* gestational age, *BMI* body mass index.

**Table 2:** Maternal and neonatal outcomes of women with and without preeclampsia.

	Without preeclampsia (n = 368)	With preeclampsia (n = 25)	p value
Gestational diabetic mellitus	34 (9.2)	7 (28.0)	0.003
Fetal growth restriction	7 (1.9)	4 (16.0)	0.003
GA at delivery (weeks)	38.1 ± 1.6	36.5 ± 2.5	0.006
**Preterm delivery**			
At GA < 37 weeks	25 (6.8)	5 (20.0)	0.016
At GA < 34 weeks	7 (1.9)	3 (12.0)	0.020
**Mode of delivery**			0.091
Vaginal delivery	148 (40.2)	9 (36.0)	
Cesarean delivery	214 (58.2)	14 (56.0)	
Forceps delivery	6 (1.6)	2 (8.0)	
Birthweight (g)	3114.4 ± 484.3	2763.6 ± 780.3	0.036
Low birth weight (2500 g)	27 (7.3)	6 (24.0)	0.004
**Apgar score**			
1 min < 7	7 (1.9)	1 (4.2)	0.401
5 min < 7	2 (0.5)	1 (4.2)	0.174
RDS	5 (1.4)	2 (8.0)	0.067
BPD	1 (0.3)	0 (0.0)	1.000
Neonatal sepsis	15 (4.1)	2 (8.0)	0.295
Perinatal death	1 (0.3)	1 (4.0)	0.123
Length of stay (days)	4.5 ± 4.9	7.0 ± 10.0	0.227

Data are presented as the mean ± SD or as N (%).

*GA* gestational age, *RDS* respiratory distress syndrome, *BPD* bronchopulmonary dysplasia.

Overall preeclamptic women and late-onset preeclamptic women had mean serum HMGB1 levels of 1112.8 ± 363.1 ng/mL and 1034.2 ± 235.6 ng/mL, respectively, which were significantly higher than normal pregnant women (910.8 ± 486.1 ng/mL) (p = 0.013 and 0.027, respectively). Early-onset preeclampsia patients had mean serum HMGB1 levels of 1280.7 ± 691.3 ng/mL, which was not statistically different from the control group (p = 0.096). Mean serum HMGB1 levels in preeclampsia women with severe feature or non-severe feature were comparable (Table 3).

**Table 3:** Serum HMGB1 levels and uterine artery Doppler findings in women with and without preeclampsia.

	Without preeclampsia (n = 368)	With preeclampsia (n = 25)	p value
**Mean HMGB1 (ng/L)**			
Overall	910.8 ± 486.1	1112.8 ± 363.1	0.013
Early-onset preeclampsia		1280.7 ± 691.3	0.096
Late-onset preeclampsia		1034.2 ± 235.6	0.027
**HMGB1 MoM**			
Overall	1.1 ± 0.6	1.4 ± 0.4	0.012
Early-onset preeclampsia		1.6 ± 0.8	0.082
Late-onset preeclampsia		1.3 ± 0.3	0.027
**Mean UAPI**			
Overall	1.0 ± 0.30	1.2 ± 0.4	0.076
Early-onset preeclampsia		1.7 ± 0.5	< 0.001
Late-onset preeclampsia		1.0 ± 0.2	0.744
Any notching (%)	46 (12.5)	8 (32.0)	0.013
Bilateral notching (%)	16 (4.3)	3 (12.0)	0.112

Data are presented as the mean ± SD, or N (%).

*HMGB1* High Mobility Group Box-1, *UAPI* uterine artery pulsatility index, *MoM* multiple of median.

There was no difference in terms of the mean UAPI between overall preeclampsia and normal groups. However, in early-onset preeclamptic women, mean UAPI was significantly higher than normal women. In the preeclampsia group, detection of an early diastolic notch on at least one side was more likely than controls (8 (32.0%) vs 42 (12.5%), p = 0.013). However, similar bilateral notching of uterine artery Doppler was observed in both groups (Table 3).

**Figure 2:**
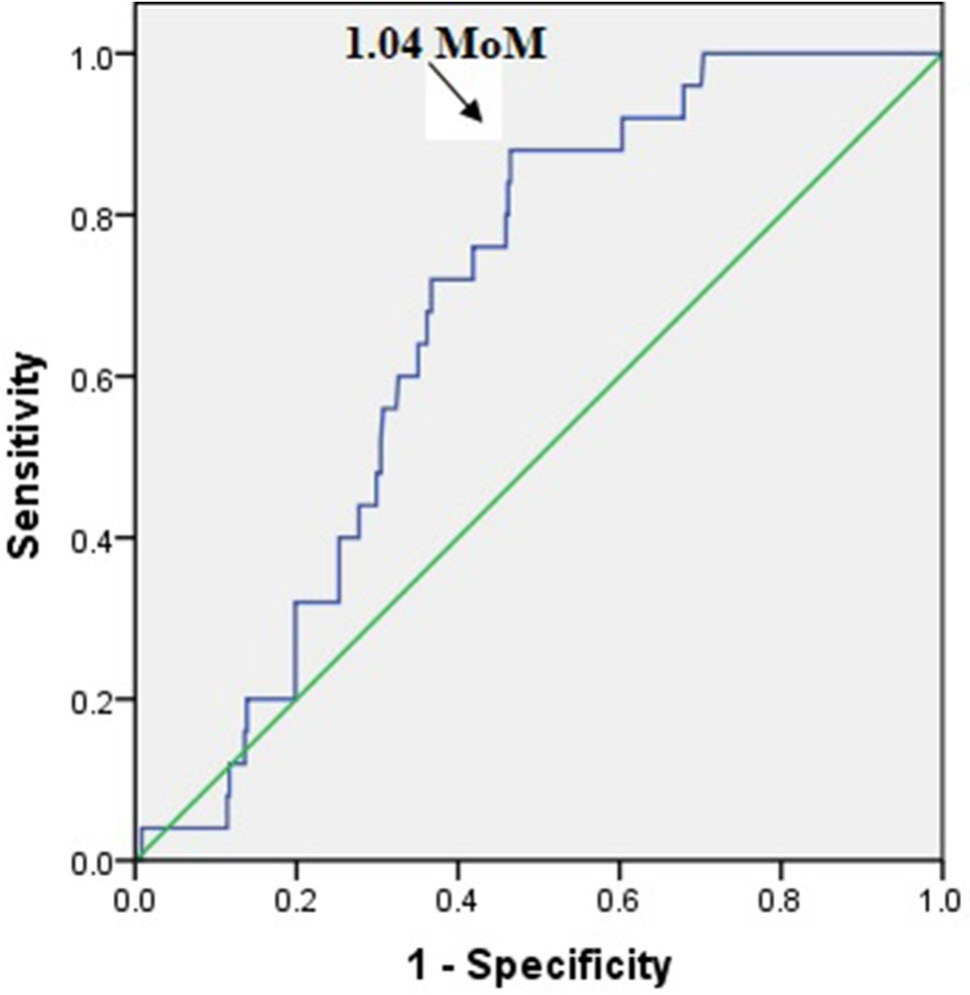
Receiver-operating characteristic curve for the relationship between serum high mobility group box-1 levels and diagnosis of preeclampsia (AUC 0.680; p = 0.003; 95% CI 0.604–0.757).

The optimum cut-off value for serum HMGB1 level at the time of measurement was 1.04 multiple of the median (MoM), according to the receiver operating characteristic curve (AUC 0.680; p = 0.003; 95% CI 0.604–0.757) (Fig. 2). The sensitivity and specificity of using serum HMGB1 levels above 1.04 MoM to predict preeclampsia were 88.0% and 53.5%, respectively. When using a mean UAPI above 95th percentile, the sensitivity and specificity for preeclampsia prediction were 8.0% and 95.1%, respectively. When using abnormal serum HMGB1 levels combined with mean UAPI to predict preeclampsia, the sensitivity and specificity were 88.0% and 50.8%, respectively (Table 4).

**Table 4:** Predictive value of serum HMGB1 levels and uterine artery Doppler for preeclampsia.

	Sensitivity (%)	Specificity (%)	PPV (%)	NPV (%)	Positive LR	Negative LR
HMGB1 levels > 1.04 MoM	88.0 (68.8–97.5)	53.5 (48.3–58.7)	11.4 (9.7–13.4)	98.5 (95.8–99.5)	1.9 (1.6–2.3)	0.2 (0.1–0.7)
Mean UAPI > 95th percentile	8.0 (1.0–26.0)	95.1 (92.4–97.1)	10.0 (2.7–31.1)	93.8 (93.1–94.5)	1.6 (0.4–6.7)	1.0 (0.9–1.1)
HMGB1 levels > 1.04 MoM and/or mean UAPI > 95th percentile	88.0 (68.8–97.5)	50.8 (45.6–56.0)	10.8 (9.2–12.7)	98.4 (95.6–99.5)	1.8 (1.5–2.1)	0.2 (0.1–0.7)

*HMGB1* High Mobility Group Box-1, *UAPI* uterine artery pulsatility index, *PPV* positive predictive value, *NPV* negative predictive value, *LR* likelihood ratio, *MoM* multiple of median.

An abnormal value of serum HMGB1 levels and an abnormal uterine artery Doppler in the second-trimester were not associated with gestational diabetes mellitus, FGR, preterm birth, low birth weight and perinatal morbidity and mortality (Table 5).

**Table 5:** Serum HMGB1 levels and uterine artery Doppler for pregnancy complications.

	Relative risk	95% Confidence interval
Preterm delivery (GA < 37 weeks)	1.0	0.7–1.4
Fetal growth restriction	1.8	0.7–4.7
Gestational diabetes mellitus	1.5	1.0–2.5
Low birth weight	1.4	0.9–2.2
Respiratory distress syndrome	1.1	0.5–2.7
Neonatal sepsis	1.7	0.8–3.5
Perinatal death	1.0	0.2–3.9

*HMGB1* High Mobility Group Box-1, *GA* gestational age.

## Discussion

According to the study, serum HMGB1 levels in the second-trimester with or without uterine artery Doppler were effective in predicting preeclampsia. The sensitivity of serum HMGB1 level in combination of UAPI to predict preeclampsia was high. However, the sensitivity of combined tests was similar to using serum HMGB1 alone because the patients in this study who had abnormal UAPI were likely to have abnormal serum HMGB1. Therefore, an additional uterine Doppler evaluation to the serum marker would not be helpful for screening preeclampsia in the second-trimester.

Previously, serum HMGB1 levels were found to be increased at the time of preeclampsia diagnosis^21,22,28^. However, our study found that the serum HMGB1 levels was elevated prior to the onset of preeclampsia symptoms, making it as one of the possible serum markers that can predict preeclampsia. Pradervand et al. reported a higher level of HMGB1 in preeclampsia (2.1 ng/ml vs 1.1 ng/ml, p = 0.03) that was not related to disease severity which was similar to our study^22^. Serum HMGB1 levels were significantly higher in overall and late-onset preeclampsia than in normal pregnant women. This study found that early-onset preeclampsia had higher HMGB1 level than control, however there was not statistically difference between two group. This may be limited by the small sample of the early-onset preeclampsia.

HMGB1 has been proved to be a key factor contributing to preeclampsia as a pro-inflammatory mediator. In severe preeclampsia, there was an increase in gene expression of HMGB1 on trophoblasts as well as an increase in circulating HMGB1 levels in maternal serum. Furthermore, the HMGB1 receptor, RAGE and S100A12 protein, were also identified in placental tissue and maternal serum. Interactions between HMGB1, RAGE, and S100A12 activate the intracellular transcriptional factors, nuclear factor-κB (NF-κB), leading to the production and release of pro-inflammatory cytokines^21,28^. HMGB1 can induce placental inflammation by production of IL-8, an inflammatory cytokine, via TLR4 on syncytium^20^. HMBG1 was also shown to increase endothelial cell permeability, which is a hallmark of endothelial dysfunction in preeclampsia^23,29^. However, HMGB1 have been shown to be associated with various inflammatory pathogenic conditions of pregnancy such as preterm premature rupture of membrane, intra-amniotic infection, recurrent pregnancy loss, maternal medical disease as well as normal pregnancy-associated mechanism such as parturition. Because of this limitation, using HMGB1 as a single screening test for preeclampsia may be problematic^30^.

In this study, pregnant women with active medical conditions and immune system disease were excluded because recent study has highlighted a close association between HMGB1, chronic inflammation, and autoimmune diseases^31^. Serum level of HMGB1 was elevated in patients with the antiphospholipid syndrome^32^.

In this study, we did not find a correlation between serum HMGB1 levels and gestational diabetes. Heim et al. reported that in hyperglycemic conditions, trophoblast increased HMGB1 secretion and triggered IL-8 via TLR4. As a result, patients with gestational diabetes may be at a higher risk of developing preeclampsia^33^.

According to this study, early-onset preeclampsia had a significantly greater mean UAPI than controls, and the presence of any early-diastolic notching was associated with preeclampsia-complicated pregnancy. While the UAPI above the 95th percentile had a high specificity for predicting preeclampsia, it had a low sensitivity. The findings were consistent with previous studies^34,35^. From the previous meta-analysis, Cnossen et al. found that an increased UAPI with notching was the best predictor of preeclampsia (positive likelihood ratio 7.5 among low-risk patients). However, in low risk populations, the sensitivity for predicting preeclampsia with UAPI alone was 42%, whereas the presence of any notching produced a higher sensitivity of 74%^36^. In contrast to our study, Dash et al. found high sensitivity of 92.6% and specificity of 84.7% for predicting preeclampsia in the second-trimester using an abnormal UAPI greater than 1.32^37^. The ability of UAPI to predict preeclampsia differs between studies and ranges from 24 to 89%. It was difficult to compare between the studies due to differences in the populations studied, gestational age at examination, Doppler technique, and the definition of abnormal flow velocity waveform^12^. Previous studies found that combined maternal serum marker with UAPI in second-trimester screening can improved the detection of early-onset preeclampsia^34,38,39^.

The strength of this study was that it is the first prospective study of HMGB1 and uterine artery Doppler in the second-trimester for preeclampsia prediction. In addition to a fetal anatomical scan at this time, evaluating the risk of preeclampsia may enable physicians to close monitoring on patients who are at high risk for preeclampsia. If a better understanding of the rise of HMGB1 in early pregnancy before clinical preeclampsia occurs, further research into intervention or prevention strategies could be possible. The limitation of the study was that it was performed during the second-trimester, when aspirin administration for preeclampsia prevention would be less effective. This combined test may have benefit in late booking antenatal care pregnant women. Identification of pregnant women at high-risk can improve pregnancy outcomes, either by early and frequent surveillance or the consideration to start acetylsalicylic acid (ASA) in high-risk patients. The United States preventive services task force recommends that women with any high-risk factors for preeclampsia should receive a low dose of ASA (81 mg/day) for preeclampsia prophylaxis administered between 12 and 28 weeks of gestation^40^. Second, because uterine artery Doppler assessment is operator dependent and subjectively interpreted, the operator's experience may have an impact on the study's findings. Third, serum HMGB1 levels may rising non-specifically to preeclampsia as discussed above, therefore interpretation should be cautious, and HMGB1 should not be used as a single screening test for predict preeclampsia. Further research into the use of serum HMGB1 levels to predict preeclampsia during the first trimester or in combination with other tests to improve the predictive value of preeclampsia would be interesting. Fourth, the cost-effectiveness and the availability of this investigation especially in low resource settings were not evaluated.

## Conclusion

This study showed that serum HMGB1 at 16–20^+6^ weeks of gestation were effective in predicting preeclampsia. The addition of UAPI did not improve the prediction performance.

## Data Availability

The datasets generated during and/or analyzed during the current study are not publicly available due to the permission of the Internal Review Board but are available from the corresponding author on reasonable request.
